# Histone Nuclear Import and Beyond: Multifunctional Roles of Importins

**DOI:** 10.1002/bies.70119

**Published:** 2026-02-24

**Authors:** Natalia Bernardes, Yuh Min Chook

**Affiliations:** ^1^ Department of Pharmacology University of Texas Southwestern Medical Center Dallas Texas USA

## Abstract

The separation of DNA‐based processes from cytoplasmic protein synthesis demands precise and effective nuclear import of histones and chromatin regulators. Because histones are highly basic and aggregation‐prone, their proper folding, sequestration, and deposition into chromatin depend on coordinated action of histone chaperones and nuclear import receptors. This review summarizes recent advances in understanding the mechanisms of core and linker histone import and chaperoning. Structural and biochemical studies have elucidated how Importin‐4/Kap123 mediates nuclear import of H3–H4 heterodimers in concert with ASF1, revealing Importin‐4's dual role as both transporter and histone chaperone. Likewise, Importin‐9/Kap114 recognizes and imports H2A–H2B heterodimers through a mechanism unusually insensitive to RanGTP, which cooperates with Nap1 for histone release. Finally, new structural analyses of the Importin‐β‐Importin‐7 heterodimer clarify its mode of linker histone H1 import. Together, these studies establish importins as multifunctional factors that couple histone stabilization, protection from aberrant interactions, nuclear import, and targeted delivery for nucleosome assembly. Outstanding questions include how secondary importins, histone modifications, and compartment‐specific chaperone dynamics regulate histone trafficking, and whether importins themselves function in nucleosome assembly. Addressing these questions will define how nuclear import integrates with chromatin homeostasis.

## Graphical abstract

Efficient nuclear import of histones requires coordinated action between histone chaperones and nuclear import receptors. Importin‐4, Importin‐9, and Importin‐β/7 not only transport specific histone complexes but also act as chaperones, stabilizing histones and ensuring their protected delivery for nucleosome assembly, highlighting importins as central regulators of chromatin homeostasis.

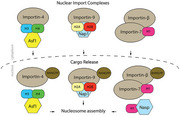

## 1 Introduction

In eukaryotic cells, the double‐membrane nuclear envelope separates DNA‐related processes in the nucleus from other cellular activities in the cytoplasm, enhancing both efficiency and control of spatially distinct processes. This compartmentalization relies on two key features of nuclear organization: DNA must be compacted to fit within the nucleus yet remain accessible to processing and regulatory machineries, and DNA‐binding proteins must be efficiently trafficked across the nuclear envelope while being protected from nonspecific interactions.

**FIGURE 1:**
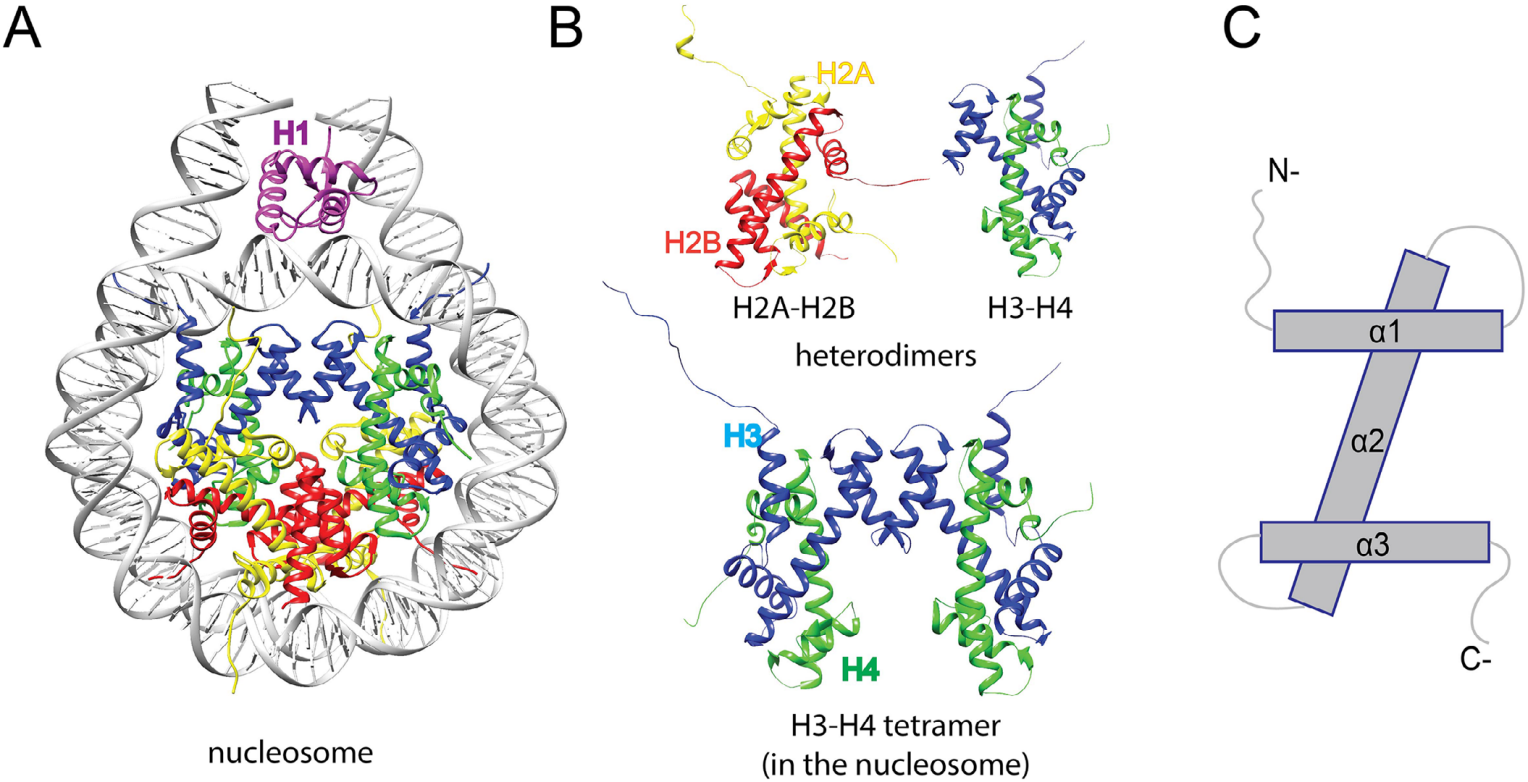
Structures of the nucleosome and histones. The nucleosome core particle (PDB 5NL0) [20] (A) is formed by (B) one tetramer of H3–H4 (blue and green cartoons, respectively) and two H2A–H2B (yellow and red, respectively) heterodimers, wrapped around ∼147 bp of DNA (gray). Linker histone H1 (pink) binds to the linker DNA emerging at both ends of the nucleosome. (C) A generic representation of the core histone‐fold domains of H2A, H2B, H3, and H4, each containing alpha‐helices α1, α2, and α3 that form the central histone‐fold domain, and the long N‐ or C‐terminal tails.

DNA compaction begins with the formation of the nucleosome, the basic unit of chromatin. Each nucleosome core consists of ∼147 base pairs of DNA wrapped around an octamer of four core histone proteins H2A, H2B, H3, and H4 (Figure 1A) [1]. Core histones are small, highly basic proteins that form H3–H4 and H2A–H2B heterodimers, each containing a conserved globular domain and disordered N‐ and C‐terminal tails (Figure 1B,C) [1]. Histone dynamics are further regulated by posttranslational modifications (PTMs) such as acetylation, methylation, phosphorylation, and ubiquitination, most of which occur on the N‐terminal tails. These PTMs modulate histone interactions with other proteins, influencing nucleosome assembly, DNA accessibility, and thereby transcription, replication and DNA repair [2].

During S‐phase, core histones are synthesized at high rates to meet the demand for nucleosome assembly following DNA replication [3]. Canonical histones H2A, H2B, H4, H3.1, and H3.2 are expressed only during S‐phase, whereas histone variants are expressed throughout the cell cycle [4]. In de novo nucleosome biogenesis, two H3–H4 heterodimers are first deposited onto replicating DNA to form a tetrasome [5], followed by incorporation of two H2A–H2B heterodimers to complete the nucleosome core [4]. Linker histone H1 binds to DNA entry and exit sites on the nucleosome and promotes higher‐order chromatin folding (Figure 1A) [6, 7]. Nucleosome recycling during replication involves segregation and reuse of parental histones, a process that differs somewhat from de novo assembly [8].

Because histones are highly basic, they are prone to nonspecific interactions and aggregation [1]. From synthesis in the cytoplasm to deposition in the nucleus, histones are therefore continuously shielded by histone chaperones. Histone chaperones are diverse proteins with different folds, and they interact with histones in different ways. These chaperones prevent aberrant interactions, promote correct folding and dimerization, and/or coordinate nuclear import and nucleosome assembly [9, 10]. In the cytoplasm, heat–shock chaperones assist in histone folding, after which H3–H4 passes through multiple histone chaperones before being transported into the nucleus, where they are transferred to nuclear chaperones [4, 11, 12]. Among these chaperones, ASF1 (Anti‐Silencing Function 1) seems to be the major chaperone that accompanies H3–H4 from the cytoplasm to the nucleus [13, 14]. Less is known about the fate of newly synthesized H2A–H2B, but in the budding yeast, the chaperone Nap1 (Nucleosome assembly protein 1) appears to accompany this heterodimer from the cytoplasm into the nucleus and possibly to their deposition onto tetrasomes [15, 16]. Canonical histone chaperones cannot permeate the NPC barrier and thus cannot import histone into the nucleus. However, cellular, biochemical, and structural evidence suggest that ASF1 and Nap1 cooperate with importins during histone import. As discussed below, it is still unclear if canonical histone chaperones make import more efficient or simply bind histones in ways that are compatible with histone–importin interactions.

Once in the nucleus, H3–H4 is directed to distinct chaperones for replication‐coupled nucleosome assembly or replication‐independent deposition [4, 17]. During transcription and DNA repair, nucleosomes are transiently disassembled to allow DNA access. Histones are removed in the reverse order of assembly, H2A–H2B dimers first, then H3–H4 tetramers, by histone chaperones [12, 18].

Efficient nucleosome biogenesis thus depends on a large and coordinated influx of histones and their chaperones into the nucleus. This transport is mediated by importins, which ferry histones through the nuclear pore complex (NPC) [19]. When these importins were first characterized two decades ago, evidence already suggested that they might serve as histone chaperones in addition to their canonical role as nuclear import receptors. In this review, we summarize the molecular players involved in the nuclear import of core histones, focusing on importins and histone chaperones specific to each core histone. In this review, we highlight structural insights of these interactions, discuss the dual functions of importins as transporters and chaperones, and examine how histones are released from importins in the nucleus for assembly into nucleosomes.

## 2 Core Histones Are Transported by Karyopherin‐β Family Nuclear Import Receptors

The high rates of histone synthesis during S‐phase necessitate active and efficient transport of newly synthesized histones into the nucleus. This import is mediated by importins, members of the Karyopherin‐β (Kap) family of nuclear transport receptors that shuttle cargoes from the cytoplasm into the nucleus [19]. Importins are flexible, negatively charged, solenoid‐shaped proteins composed of 20–24 tandem HEAT repeats, each formed by a pair of α‐helices connected by short loops, with occasional extended loops [19, 21, 22]. These structural features enable importins to bind both cargoes and nucleoporins (proteins forming the NPC), allowing Importin–cargo complexes to traverse the NPC permeability barrier [23, 24].

Transport directionality is controlled by the small GTPase Ran, which is asymmetrically distributed as RanGTP in the nucleus and RanGDP in the cytoplasm [25]. Importins bind cargoes and RanGTP in a mutually exclusive manner: they associate with cargo in the RanGTP‐depleted cytoplasm and release them in the RanGTP‐rich nucleus, ensuring vectorial transport [26, 27].

Many protein cargoes carry nuclear localization signals (NLSs), short sequence motifs that mediate importin binding. The various NLS classes and their cognate importins have been reviewed elsewhere [28, 29]. Recent work, however, has shown that importins also recognize nonlinear or structured motifs. Some use globular WW domains to bind an IMPα loop, while others use modular folded domains that contact the concave surfaces of importins [30, 31, 32, 33, 34, 35, 36].

Importin–core histone complexes form through this latter mode of interactions: the histone‐fold domains of H3–H4 and H2A–H2B heterodimers binding directly to their respective importins [30, 35].

## 3 Importin‐4 Is a Key Nuclear Importer of Histones H3 and H4

### 3.1 Insights From Cellular and Biochemical Studies

**FIGURE 2:**
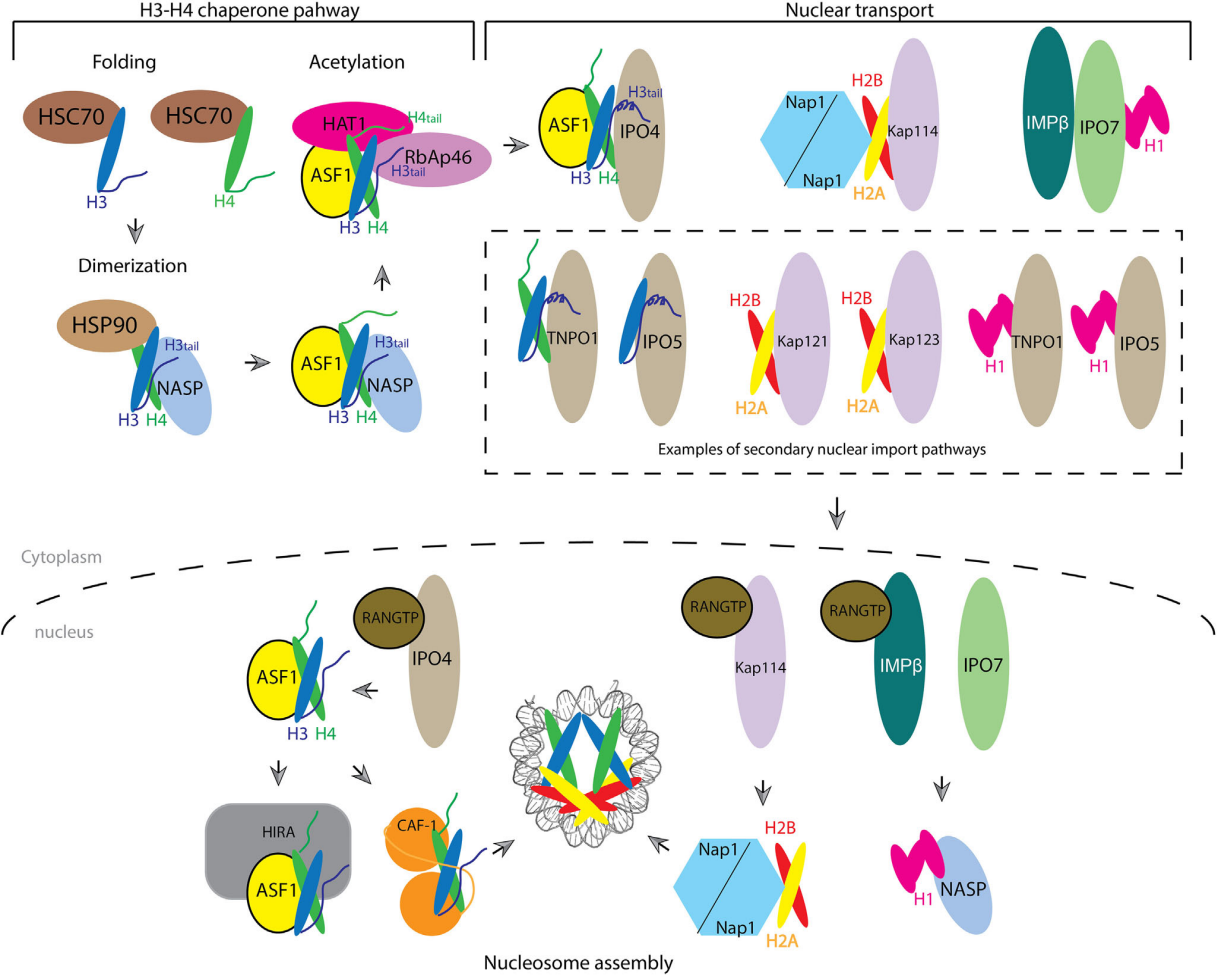
Schematic of the H3 and H4 histone chaperone pathway and nuclear import of histones. After protein synthesis in the cytoplasm, H3, and H4 are folded and dimerized by heat–shock proteins HSC70 and HSP90. NASP stabilizes the H3–H4 heterodimer, transfers it to ASF1 and the acetyltransferase complex HAT1‐RBAP46 to promote histone acetylation. Importin‐4 (Imp4) then binds to form the ASF1–H3–H4–Imp4 nuclear import complex. Other potential secondary nuclear import receptors are TNPO1 and Importin‐5 (Imp5). Once in the nucleus, the binding of Importin‐4 to RanGTP likely triggers release of ASF1–H3–H4. ASF1 will then hand off H3–H4 to CAF‐1 or HIRA for deposition into assembling nucleosomes. Less is known about the chaperone pathway of histones H2A, H2B, and H1. In yeast cells, Nap1 is the major histone–chaperone that interacts with H2A–H2B in the cytoplasm and nucleus. Nap1 forms a stable complex with Kap114–H2A–H2B. RanGTP binds Nap1–Kap114–H2A–H2B and reorients the bound H2A–H2B, facilitating its transfer from the importin to Nap1 and then to the assembling nucleosome or a nuclear chaperone. Histone H1 binds the Impβ–Imp7 heterodimer in the cytoplasm. In the nucleus, RanGTP binding to Impβ, perhaps with the help of histone–chaperone NASP, releases H1.

Biochemical analyses of histone H3.1/H3.3 and histone chaperone ASF1 complexes purified from HeLa cell cytoplasm revealed a cascade of histone chaperone interactions that escort human H3 and H4 from synthesis to nuclear import [11, 37, 38]. Newly synthesized histones are first folded by HSC70, followed by interactions with HSP90 and NASP, which promote H3–H4 dimerization (Figure 2) [11, 37]. The NASP–H3–H4 complex may then associate with ASF1 to form a quaternary complex NASP–H3–H4–ASF1 assembly [39, 40]. NASP is subsequently exchanged for HAT1/RbAp46 complex, which acetylates H4 at K5 and K12 (Figure 2) [11, 38, 41, 42, 43]. Finally, ASF1–H3–H4 is transferred primarily to Importin‐4, and to a lesser extent Importin‐5, for nuclear import (Figure 2) [11, 37]. Other studies suggest that additional factors like Codanin‐1 and MMS22L bind ASF1 to restrict premature histone release, coordinating Importin‐4‐mediated nuclear import of ASF1–H3–H4 with S‐phase histone demand, via a mechanism that is not understood [44, 45].

In the nucleus, RanGTP binding to Importin‐4 likely triggers release of ASF1–H3–H4, enabling ASF1 to hand off H3–H4 to CAF‐1 for replication‐coupled deposition or to HIRA or DAXX for replication‐independent assembly (Figure 2) [11, 12, 46].

Complementary studies in yeast and human cells identified the Kaps responsible for histone import [47, 48, 49]. Yeast cell extracts revealed Kap123 as the major H3/H4 binder [48] while cytosolic extracts from human cells similarly identified its homolog Importin‐4 [11, 37, 38]. Recombinant binding assays confirmed that Importin‐4 can bind directly to H3 and/or H4 [49, 50]. In addition to this canonical role of core histone import, a testis‐specific Importin‐4 ortholog in *Drosophila*, APOLLO, was found to mediate import of the protamine‐like protein Mst77F or TP2 (Transition Protein 2) in rat, during spermatid chromatin remodeling, indicating that Importin‐4 also functions in additional chromatin contexts beyond somatic histone transport [51, 52].

**FIGURE 3:**
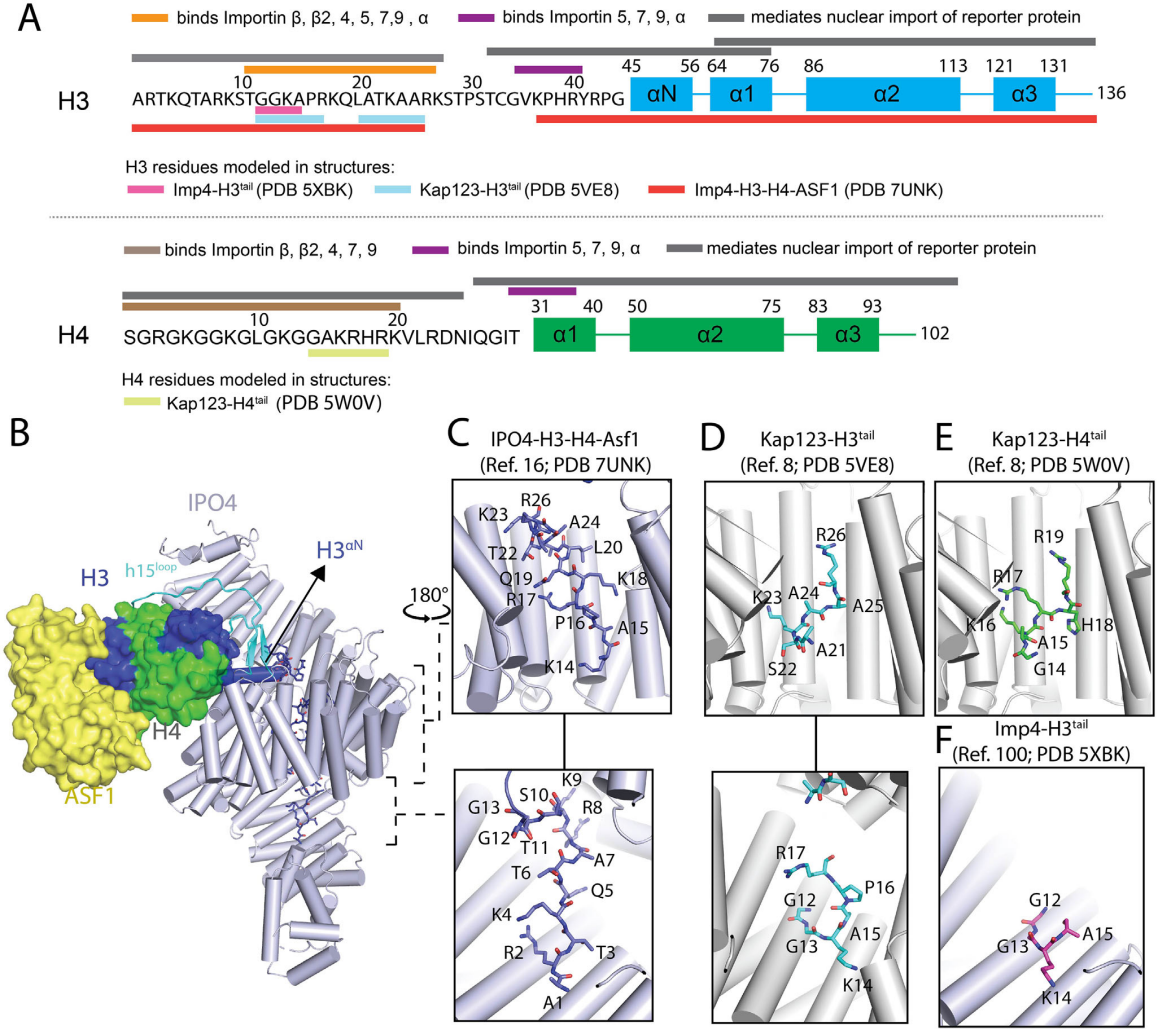
Interaction of importins with H3–H4. (A) Representations of the H3 and H4 polypeptides, showing the sequences of their N‐terminal tails and the secondary structural elements of their central core domains. The colored bars above the sequences summarize previous studies showing histone segments that bind various importins, and those that can mediate the nuclear import of a fused reporter protein [53]. The colored bars below the sequences mark the histone segments observed to bind importins in four different structures. (B) Cryo‐EM structure of Importin‐4 bound to H3–H4–ASF1 [30], with zoomed in views of the H3 tail binding sites in inserts on the right (C). (D–F) Four panels show the same regions as the inserts in (C) from the following crystal structures: (D) Two panels show H3 tail Residues 1–28 (cyan) binding to Kap123 (gray) [57]; (E) H4 tail Residues 1–34 (green) binding to Kap123 [57]; (F) H3 tail Residues 1–18 of binding to a fragment of Importin‐4 [58].

Because the basic N‐terminal tails of H3 and H4 are enriched with basic residues and thus resemble classical NLSs, multiple groups used nuclear import and pull‐down assays with the tail fragments to map potential NLS motifs [37, 48, 53, 54]. The H3 tail (Residues 1–28) was sufficient to localize reporter proteins to both HeLa and yeast cell nuclei, and it could bind purified TNPO1, Importin‐β, ‐4, ‐5, ‐7, ‐9, and ‐α (Figure 3A) [37, 48, 53, 54, 55]. Likewise, the H4 tail fragment (Residues 1–21) localized to the nucleus and bound TNPO1, Importin‐β, ‐4, ‐7, and ‐9 (Figure 3A) [37, 54]. These results suggested that multiple importins can interact with the very basic H3 and H4 tails. Although these fragments can bind importins, they are not essential for nuclear localization of H3 and H4 as constructs of the histone‐fold domain also mediated nuclear import (Figure 3A) [53, 56]. Moreover, estimated relative affinities of H3/H4 tails for various importins did not match the cellular observation that Importin‐4/Kap123 is the predominant importer whereas the ASF1–H3–H4 complex showed a clear preference for Importin‐4, consistent with its dominant role in vivo [54]. This realization, together with advances in cryogenic electron microscopy (cryo‐EM), enabled structural insights into how Importin‐4 engages histones.

### 3.2 How Kap123/Importin‐4 Recognizes H3 and H4 for Nuclear Import

The identification of Kap123/Importin‐4 as the principal H3/H4 importer prompted structural studies to understand their mode of histone recognition. Early x‐ray crystallography of full‐length Kap123 or an Importin‐4 fragment bound to isolated H3 or H4 tail peptides modeled only a few tail residues, each binding along the inner, concave surface of the importin solenoid (Figure 3C–F) [57, 58]. The histone‐fold domains were omitted in these constructs.

Recent advances in cryo‐EM have enabled atomic‐resolution structure determination of large, dynamic protein assemblies using relatively small amounts of material. Importantly, cryo‐EM can also capture multiple conformational states within a single complex, providing insight into the dynamics and structural transitions that may be biologically relevant. For the H3–H4 system, a major advance came from a 3.5 Å resolution cryo‐EM structure of full‐length human Importin‐4 bound to yeast ASF1 and *Xenopus laevis* H3–H4, which revealed how the importin and histone chaperone co‐engage the histone heterodimer [30]. Recombinant *X. laevis* H3–H4 dimers were used for structural and biochemical studies owing to their higher stability and > 90% identity to human H3–H4, while yeast ASF1 was previously used with *X. laevis* H3–H4 to solve the crystal structure of the ASF1–H3–H4 complex [13]. The Importin‐4–H3–H4–ASF1 complex, long inferred from biochemical analysis [11, 37, 38], provided the first full‐length structure of Importin‐4.

Importin‐4 shares strong architectural similarity with Kap123: its 24 HEAT repeats (h1–h24) form a superhelix with long loops in repeats h8 and h15 (Figure 3B) [30, 57]. A notable feature is the tightly coiled superhelix forming a closed central ring stabilized by intramolecular interactions between h6–h9 and h17–h18 (Figure 3B) [30]. A similarly closed configuration occurs in Kap121 and its human homolog Importin‐5, whereas yeast Kap123 achieves closure via a C‐terminal helix [57, 59]. These similarities underscore the close evolutionary relationship between Importin‐4/Kap123 and Importin‐5/Kap121 [60].

In the Importin‐4–H3–H4–ASF1 structure, ASF1 binds H3–H4 as in its previous crystal structure [13, 30]. This structure provides the first example of histone co‐chaperoning by an importin and a canonical histone chaperone. As seen in other H3–H4 complexes, such as HAT1–HAT2–H3–H4–ASF1 [42], NASP–H3–H4–ASF1 [39], and the nucleosome [1], the H3 αN‐helix adopts distinct orientations relative to the histone‐fold domain, suggesting that conformational flexibility may facilitate histone handoff between chaperones.

## 4 Other Importins Have Minor Roles in H3 and H4 Nuclear Import

In addition to Importin‐4/Kap123, several other importins contribute to H3 and H4 nuclear import. Pull‐down assays with yeast importins and nuclear localization analyses in importin‐deficient strains identified Kap121 and Kap104 as additional importers [37, 48, 49, 54, 61]. Consistently, their human homologs Importin‐5 and TNPO1 bind H3–H4 in cytosolic extracts, particularly when Importin‐4 is depleted [11, 37]. Multiple importins can be pulled down with GST–H3 or GST–H4, although the physiological relevance of these interactions remains uncertain. Among the importins implicated, Importin‐5 and TNPO1 are the most consistently observed and likely function as secondary or backup importers for H3–H4, although the extent of their contribution to histone import remains unclear.

Although most evidence supports the import of H3 and H4 as a heterodimer, recent studies suggest that some cytosolic H3 and H4 pools remain monomeric and may heterodimerize only after nuclear entry [62, 63]. H3 and H4 species that cannot form heterodimers preferentially associate with Importin‐5 and NASP in cells [62, 63]. These observations point to multiple routes for histones, possibly reflecting distinct chaperoning or quality‐control pathways.

## 5 PTMs of the H3 and H4 Tails and Nuclear Import

Histone PTMs are covalent chemical marks that regulate histone folding, chaperoning, and chromatin assembly. Most PTMs on H3 and H4 occur on lysine and arginine residues of their N‐terminal tails. Although these tails are not essential for nuclear import [30], PTMs can influence chaperone pathways and indirectly modulate importin interactions.

The most prominent cytoplasmic PTMs are acetylation of H4 at lysines K5 and K12, consistent with the presence of HAT1–HAT2/RbAp46 bound to H4 in HeLa cell cytosolic extracts [38, 64, 65]. These modifications stabilize H4 association with ASF1 and facilitate transfer to Importin‐4, promoting efficient nuclear import, and subsequent chromatin assembly [11, 44]. H3 is also acetylated in the cytoplasm, at lysines 9, 14, and 18 [11]. Although their direct contribution to importin binding remains unclear, these PTMs may modulate chaperone engagement or restrict binding to select importins.

Biochemical studies using acetylation‐mimic mutations in H3 and H4 tails have yielded mixed results. Some reported stronger binding of acetylated H4 to Importin‐4 and more efficient nuclear import [37], whereas others observed moderate or negligible results [47], and in some cases acetylation of H3 reduced importin binding [54]. In yeast, acetylation‐mimic mutations in H3 and H4 tails do not affect cell viability [47], consistent with PTMs acting to fine‐tune rather than dictate import efficiency.

Mono‐ and di‐methylation of H3K4 and H3K9 have been detected on soluble, non‐nucleosomal histones [62, 66]. H3K4 methylation correlates with properly folded histones, whereas aberrant methylation or demethylation may mark misfolded or misprocessed intermediates [67]. Ubiquitination of H3 and H4, may regulate histone turnover, proteasomal degradation, or quality control prior to import [68, 69]. Phosphorylation of residues such as H4S47 and H3T3 has also been proposed to influence chaperone exchange dynamics and histone availability, although its cytoplasmic role remains speculative [70, 71].

Together, these studies indicate that while acetylation of H3 and H4 enhances chaperone association and indirectly facilitates nuclear import, it is not strictly required for Importin‐4 recognition. Instead, histone PTMs act as molecular checkpoints that fine‐tune chaperone and importin interactions, prevent aggregation or degradation, and ensure that only properly assembled histones are delivered to the nucleus for chromatin assembly.

## 6 Importin‐9/Kap114 Are the Main Nuclear Import Receptors for H2A–H2B

### 6.1 H2A–H2B: From Synthesis in the Cytosol to Nucleosome Assembly

Posttranslation processing and transport of histones H2A and H2B in the cytoplasm remain poorly characterized. Pull‐down experiments from yeast and HeLa cell extracts identified the histone‐chaperone Nap1 as a cytosolic binding partner of H2A and/or H2B [16, 72, 73]. Similar assays revealed that Kap114 (yeast) and its human homolog Importin‐9 are the principal importins associated with H2A and H2B [74, 75]. Nap1 and Kap114 frequently co‐purify with H2A and H2B, suggesting that they form a ternary complex during nuclear import [16, 73].

Once in the nucleus, Nap1 may contribute to nucleosome deposition together with histone chaperones FACT and/or Nucleoplasmin [76, 77]. Nucleoplasmin, first characterized in *Xenopus* oocytes, binds H2A–H2B and facilitates their deposition onto tetrasomes [78]. The FACT complex functions as a histone chaperone that reorganizes nucleosomes during transcription, replication, and repair, and can interact with H2A–H2B to promote their disassembly and exchange from nucleosomes [77, 79].

### 6.2 Other Importins as Secondary H2A–H2B Importers

As with H3 and H4, H2A, and H2B can associate with multiple importins. In *Saccharomyces cerevisiae*, Kap121 and Kap123 act as secondary import receptors that bind H2A and H2B in Δkap114 strains [61, 75]. Correspondingly, Kap121 and Kap114 null mutants display markedly impaired H2A–H2B nuclear import [61].

**FIGURE 4:**
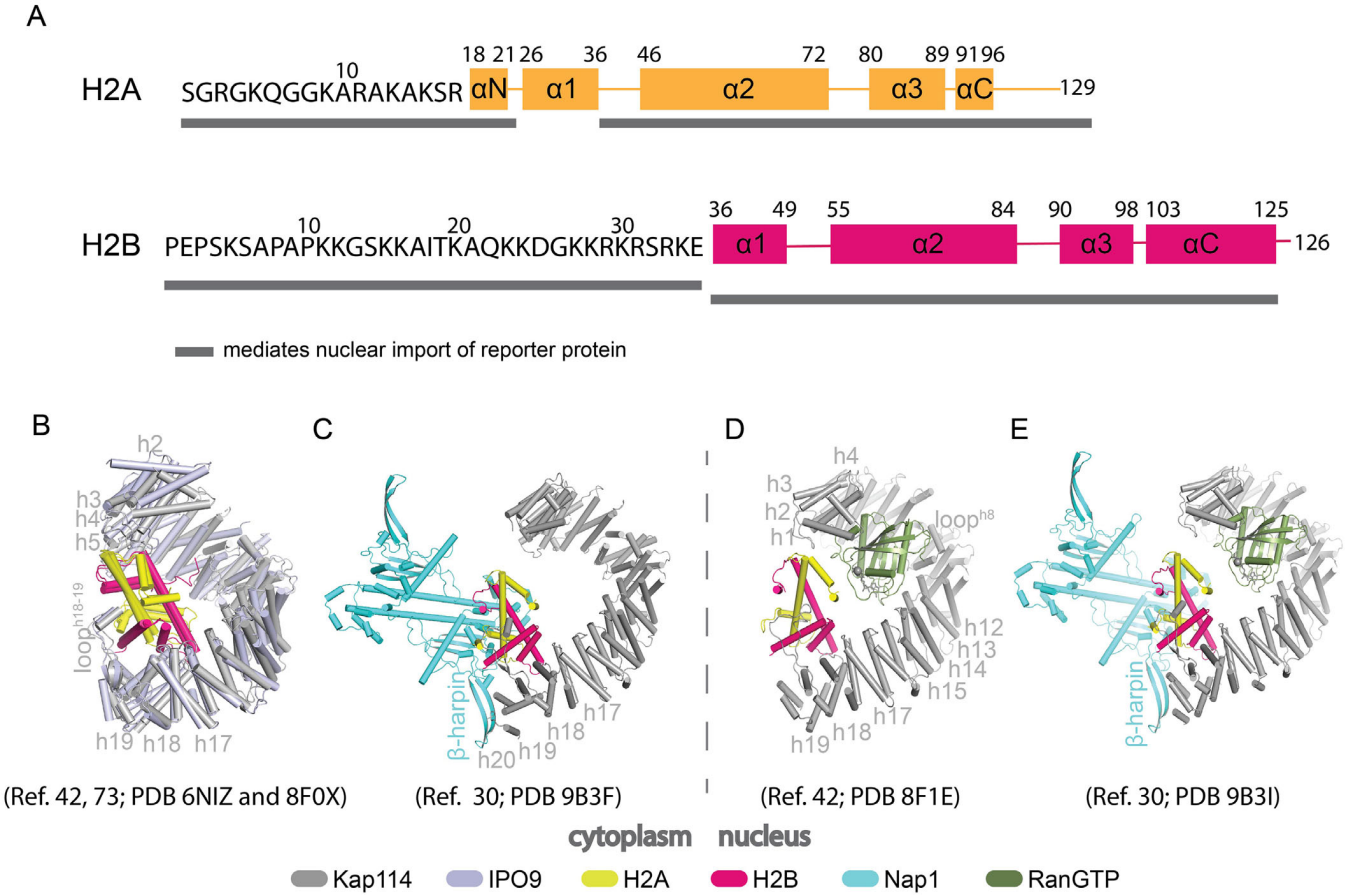
Interaction of importins with H2A–H2B. (A) Representations of the H2A and H2B polypeptides, showing the sequences of their N‐terminal tails and the secondary structural elements of their core domains. The dark gray bars mark histone segments that can mediate the nuclear import of a fused reporter protein [53]. (B) Overlayed structures of Importin‐9–H2A–H2B [35] and Kap114–H2A–H2B [81] complexes (Importin‐9/light blue, Kap114/gray, H2A/yellow, and H2B/red). (C–E) Cryo‐EM structures of Kap114–H2A–H2B–Nap1 (C; Nap1/cyan) [84]; RanGTP‐Kap114‐H2A‐H2B (D; Ran/dark green) [81], and RanGTP–Kap114–H2A–H2B–Nap1 (E) [84]. (B) and (C) lack RanGTP and may represent assemblies in the cytoplasm, while (D) and (E) contain RanGTP and may represent nuclear assemblies. In the absence of Nap1 and RanGTP (B), the N‐ and C‐terminal HEAT repeats of Importin‐9/Kap114 engage the histone‐fold domain of H2A–H2B. When Nap1 or RanGTP is bound, H2A–H2B engages only the C‐terminal repeats of the importin. Nap1's β‐hairpin binds to the importin C‐terminus, and RanGTP binds the N‐terminal repeats of the importin.

Pull‐down binding assays using immobilized histones demonstrated direct interactions of H2A and/or H2B with Kap114, Kap104, Kap123, Kap121, and Kap95 from yeast cells, and with Importin‐9 and ‐β in human cells [49, 61]. Nuclear localization studies in HeLa cells further showed that both the N‐terminal tails of H2A (Residues 1–25) and H2B (Residues 1–35) and their histone‐fold domains (H2A 37–129; H2B 36–125) can all mediate nuclear localization (Figure 4a) [53, 80].

### 6.3 Structural Analyses of Importin‐9 and Kap114 Bound to H2A–H2B

Crystal and cryo‐EM structures of human Importin‐9‐*X. laevis* H2A–H2B and yeast Kap114–H2A–H2B complexes revealed how these homologous importins recognize the histone dimer (Figure 4B) [35, 81]. *X. laevis* H2A–H2B was used for these studies because it is nearly identical to human histones while exhibiting more favorable biochemical behavior. Both importins contain 20 HEAT repeats that form open solenoid architectures encircling the histone‐fold domain (Figure 4B) [35, 81]. Prominent long loops connect helices within repeats h8 and h19, and between h18 and h19: the h8 loop mediates Ran binding, while h18–19 and h19 loops contact H2A–H2B and Nap1, respectively [35, 81]. The solenoid wraps around H2A–H2B such that the N‐terminal (h2–h5) and C‐terminal (h18–h20 + h18–19loop) repeats clamp the histone‐fold domain, shielding its DNA‐binding surfaces in a chaperone‐like fashion (Figure 4B) [35, 81].

### 6.4 H2A–H2B Is Not Released From Importin‐9/Kap114 by RanGTP

In contrast to canonical nuclear import pathways, RanGTP binding does not trigger cargo H2A–H2B release from Importin9 or Kap114. Instead, it forms the stable complexes RanGTP–Kap114–H2A–H2B or RanGTP–Importin9–H2A–H2B (Figure 4D) [35, 61, 81, 82]. Cryo‐EM and hydrogen–deuterium exchange analyses of the RanGTP–Kap114/Importin‐9–H2A–H2B complex show RanGTP bound in its canonical position, while H2A–H2B slightly reoriented: its contacts with the importin's C‐terminal HEAT repeats remain intact, but interactions with N‐terminal repeats are lost (Figure 4D,E). This rearrangement exposes the histone interfaces that engage H3–H4 and DNA in the nucleosome, thereby priming H2A–H2B transfer to assembling nucleosomes or nuclear chaperones (Figure 4D,E) [81, 82].

### 6.5 RanGTP and Nap1 Cooperate to Release H2A–H2B From Importin‐9/Kap114

Early biochemical studies showed that yeast Nap1 interacts with H2A–H2B–bound Kap114 in both cytoplasmic and nuclear extracts, implying that Nap1 functions as a co‐import factor and later facilitates histone release for nucleosome assembly [16, 73, 75, 83]. A recent cryo‐EM structure of the Nap1–H2A–H2B–Kap114–RanGTP complex revealed how Nap1 and RanGTP cooperate to chaperone and release the histone dimer (Figure 4C,E) [84]. The histone in this quaternary complex is sandwiched between Kap114 and the Nap1 dimer, while RanGTP binds the importin's N‐terminal repeats, as in the ternary RanGTP–Kap114–H2A–H2B complex (Figure 4E). Comparison with the binary Kap114–H2A–H2B structure suggests that Nap1 is positioned to shield H2A–H2B as it is transferred away from the importin to downstream nuclear chaperones or assembling nucleosomes (Figure 4E).

In contrast to the Ran‐containing complex, which adopts a single defined architecture, the Nap1–Kap114–H2A–H2B assembly in the absence of RanGTP populates multiple alternative arrangements, presenting challenges for high‐resolution structure determination. These include configurations in which H2A–H2B engages Kap114 alone while Nap1 associates with the importin without contacting the histone, as well as assemblies in which the histone dimer is positioned between Kap114 and the Nap1 dimer and interacts with both proteins. The coexistence of these discrete states points to a dynamic interaction landscape rather than a single static architecture. Rather than reflecting on nonspecific heterogeneity, these alternative arrangements likely represent functionally relevant intermediates that facilitate histone shielding and stepwise transfer during nuclear import and handoff.

Notably, although the mode of Nap1 engagement in the yeast Nap1–H2A–H2B–Kap114 complex is conserved among other yeast and human Nap1–H2A–H2B structures [15, 85], it differs from the binding modes reported in other organisms. In *Caenorhabditis elegans*, H2A–H2B associates with only one subunit of the Nap1 dimer, whereas in *Arabidopsis thaliana* NRP1 the histone dimer engages a nonconserved acidic region within the dimerization helix [86, 87, 88]. Together, these observations highlight a conserved Nap1 scaffold that supports diverse, species‐specific modes of H2A–H2B engagement.

## 7 The Importin‐7‐Importin‐β Heterodimer Transports Linker Histone H1 Into the Nucleus

### 7.1 The Chaperoning System for Linker Histone H1

The pathway for chaperoning and importing linker histone H1 remains incompletely understood, largely because of the dynamic nature of H1‐chaperone complexes. Histone chaperones tNASP, sNASP, TAF‐1, and Protα co‐immunoprecipitate with H1 from HeLa and mouse cell extracts, with tNASP also copurifying with HSP90, suggesting a role for HSP90 in H1 folding in the cytoplasm prior to its handoff to tNASP [89, 90, 91, 92, 93].

In *Xenopus* egg extracts, Nap1 and Nucleoplasmin are also associated with H1 [76, 94, 95]. In vitro chromatin reconstitution assays indicate that NASP, NAP1, or TAF‐1 can facilitate H1 deposition onto linker DNA [89, 92, 94, 95], whereas ProTα and Nucleoplasmin promote H1 displacement from chromatin [76, 91, 96]. Pull‐down experiments further identified Importin‐β, ‐α, and ‐7 as H1‐interacting partners in HeLa cell extracts [97], prompting deeper investigations into how these importins chaperone and transport H1 into the nucleus.

### 7.2 Interactions of H1 With Multiple Importins

In vitro binding assays using immobilized full‐length H1 with recombinant Importins revealed that human H1 binds Importin‐β and Importin‐7, as well as Importin‐5 and Transportin‐1 [98]. Notably, while Importin‐β and ‐7 can each bind H1 individually, the Importin‐β–Importin‐7 heterodimer binds with substantially higher affinity, and both importins are required for efficient H1 nuclear accumulation in cells [97, 98].

**FIGURE 5:**
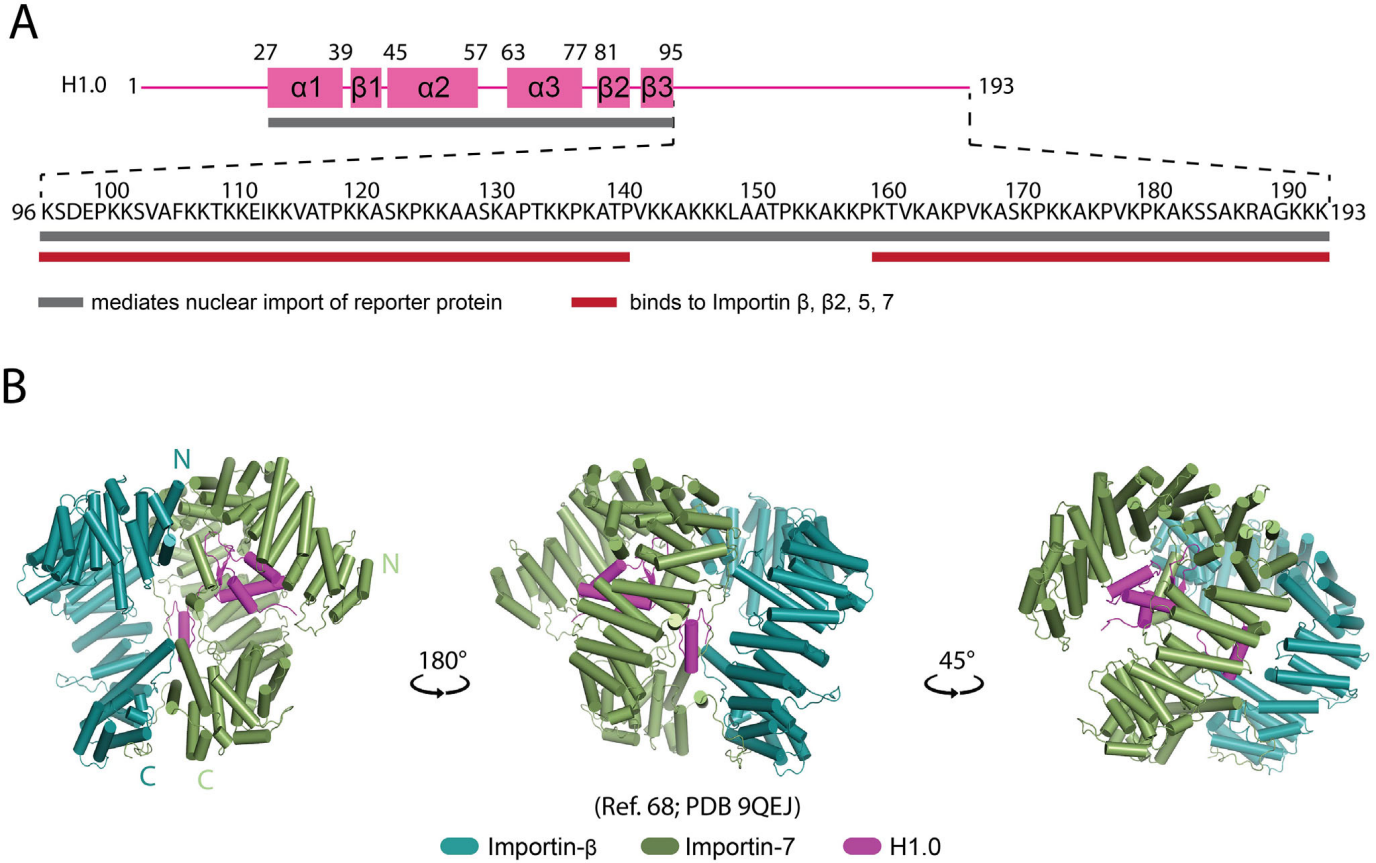
Interactions of linker histone H1 with importins. (A) Diagram showing the secondary structural elements of histone H1.0 and the sequence of its C‐terminal tail. Gray bars indicate H1 fragments that mediate the nuclear import of a fused reporter protein, and red bars show fragments that bind to various importins [53, 98]. (B) The revised PDB model of the Importin‐β–Importin‐7–H1 complex [102].

H1 consists of a short N‐terminal tail, a central winged‐helix globular domain, and a long C‐terminal tail—a domain organization distinct from that of core histones (Figure 5A) [99, 100]. Nuclear localization mapping showed that both fragments encompassing its C‐terminal tail (Residues 95–193) or globular domain have nuclear targeting capabilities [53, 101]. C‐terminal fragments bound Importin‐5, ‐β, ‐7, and Transportin‐1, but only the Importin‐β–Importin‐7 heterodimer efficiently imported the full C‐terminal tail [98]. These findings established Importin‐β–Importin‐7 as the principal nuclear import receptor of linker histone H1.

### 7.3 Structures of H1 Bound to the Importin‐β–Importin‐7 Heterodimer

A low‐resolution (6.2 Å) cryo‐EM map of the Importin‐β–Importin‐7–H1 complex provided the first structural insight into how the importin heterodimer recognizes human H1, showing a cage‐like conformation that encloses the histone's globular domain (Figure 5B) [33]. This initial model (PDB 6N88) placed the H1 globular domain (Residues 22–95) at the concave surface of Importin‐β, with the H1 C‐terminal tail contacting Importin‐7 [33].

Subsequent AI‐guided modeling (AlphaFold 3) combined with cross‐linking data revised this arrangement: Importin‐7, not Importin‐β, binds the H1 globular domain (PDB 9QEJ) [102]. In this updated model, Importin‐β and ‐7 (each composed of 19 HEAT repeats) associate through their C‐terminal HEAT repeats to form an extended U‐shaped superhelix (Figure 5B) [33, 102]. The acidic loop linking the h19 helices of Importin‐7 mediates extensive contacts between the two importins, while the disordered C‐terminal tail of Importin‐7 engages hydrophobic pockets in Importin‐β’s C‐terminal repeats [102]. The globular domain of H1 binds near the RanGTP binding site of Importin‐7, and the H1 C‐terminal tail may thread between the two importins [33]; however, the poor quality of the map suggests that the placement of the tail in this region is uncertain [102]. This binding mode implies that RanGTP binding to Importin‐β in the nucleus promotes its dissociation from Importin‐7–H1 complex by displacing the C‐terminal region of Imp7 that binds to Impβ [102]. The subsequent release of H1 from Importin‐7 remains unresolved; either RanGTP may directly displace H1 from Importin‐7, or Nasp binding could assist in disassembly and nucleosome deposition [97, 102].

## 8 Summary

Importins actively transport both canonical and linker histones from the cytoplasm into the nucleus. In human cells, Importin‐4, Importin‐9, and the Importin‐β–Importin‐7 heterodimer are the primary nuclear import receptors for H3–H4, H2A–H2B, and H1, respectively. Additional importins such as Importin‐5 and TNPO1 may act as backup or context‐specific importers, possibly recognizing histone variants or PTM forms.

Beyond transport, Importins also function as histone chaperones, shielding highly basic histones from nonspecific interactions en route to nucleosome assembly. Cryo‐EM structures have revealed that Importin‐4 wraps the H3 N‐terminal tail and contacts the H3–H4 globular domain of H3–H4 (with ASF1); Importin‐9 encapsulates the H2A–H2B fold; and the Importin‐β–Importin‐7 heterodimer jointly chaperones the globular domain and the C‐terminal tail of H1.

Importin‐9 also participates in nucleosome assembly through interactions with Nap1 and RanGTP, facilitating H2A–H2B transfer onto tetrasomes. Despite recent structural advances, major questions remain regarding how backup importins recognize histones, how histone variants or PTMs influence import pathways, the potential roles of importins in nucleosome assembly, and the mechanism of H1 release in the nucleus.

## 9 Outstanding Questions About Nuclear Import of Histones and Beyond

### 9.1 What Are the Roles of Secondary (“Backup”) Histone Importers?

Roughly one‐third of all nuclear import cargoes can bind multiple importins, and histones exemplify this redundancy [103]. The specific importin–cargo pairing likely depends on relative affinities, expression levels, and cellular context. For histones, the roles of secondary importers such as Importin‐5/Kap121 and Transportin‐1 remain unclear. Differences in histone folding state (H3–H4 dimers vs. monomers), PTMs, or variant sequences may modulate importin affinity and route selection. Combined structural and biochemical studies of importins bound to distinct histone types will be critical to dissect this regulation.

### 9.2 Do Importins Function in Nucleosome Assembly or Disassembly?

The discovery that Importin‐9/Kap114 can cooperate with RanGTP and Nap1 to deliver H2A–H2B to DNA suggests nuclear import receptors may have a role in nucleosome assembly [16, 35, 81, 84]. Similarly, Importin‐4's strong binding to H3–H4–ASF1 (*K*_D_ ∼ 30;gt tnM; [30]) and its cochaperone role points to possible nuclear functions. Although RanGTP can dissociate histone tails from importins [54], it is unclear whether this applies to full‐length H3–H4–ASF1. Further structural and biochemical studies on Importin‐4–H3–H4–ASF1 in the presence of RanGTP and DNA, combined with proteomics analyses of nuclear importins, could clarify these roles.

### 9.3 Can Importins Regulate Histones PTMs?

The timing and sequence of PTM addition and removal during histone maturation remain poorly defined. Given that importins sequester histones prior to nuclear entry, they may influence histone accessibility to modifying enzymes such as HAT1/RbAP46, thereby serving as regulatory checkpoints that coordinate PTM state with nuclear import.

### 9.4 Where Do Chaperone‐Histone Interactions Occur—Cytoplasm or Nucleus?

One fundamental unresolved question concerns the cellular compartments in which specific histone–chaperone interactions occur. Current models rely on analyses of fractionated extracts, which are prone to nuclear leakage and cross‐contamination. More precise methodologies are needed to map the sequence of chaperoning events from histone synthesis to deposition into nucleosomes.

## Author Contributions

The authors take full responsibility for this article.

## Conflicts of Interest

The authors declare no conflicts of interest.

## Data Availability

Data sharing not applicable to this article as no datasets were generated or analyzed during the current study.
